# Emerging Multiorgan Klebsiella pneumoniae Invasive Syndrome Leading to Septic Shock: A Case Report and Review of the Literature

**DOI:** 10.7759/cureus.26647

**Published:** 2022-07-07

**Authors:** Diana M Villanueva, Pardeep Taunk, Padmanabhan Krishnan, Nilka Figueroa

**Affiliations:** 1 Department of Internal Medicine, Coney Island Hospital, Brooklyn, USA; 2 Department of Pulmonary and Critical Care Medicine, Coney Island Hospital, Brooklyn, USA; 3 Department of Infectious Diseases, Coney Island Hospital, Brooklyn, USA

**Keywords:** ophthalmic septic emboli, klebsiella pneumoniae (kp), necrotizing pneumonia, liver abscess drainage, emerging disease, emerging pathogen, septic shock, hypervirulent klebsiella pneumoniae, klebsiella pneumoniae invasive syndrome (kpis), invasive liver abscess syndrome (ilas)

## Abstract

*Klebsiella pneumoniae* *(K. pneumoniae)* is a community-acquired pathogen that typically causes pneumonia and urinary tract infections. Rarely, it can affect other organ systems such as the gastrointestinal tract, as well as the meninges, ears, eyes, and spine. We present the case of a 62-year-old male admitted with septic shock secondary to necrotizing pneumonia and multiple hepatic liver abscesses, which to the best of our knowledge, is the first reported case of multiorgan invasive *K. pneumoniae* infection, including the presence of a newly recognized syndrome referred as Invasive Liver Abscess Syndrome (ILAS). It is important to maintain both ILAS and *K. pneumoniae* invasive syndrome (KPIS) in the differential diagnosis, especially in areas of the world with a large number of tourists and immigrants, such as New York City, where the presented case took place.

## Introduction

Invasive liver abscess syndrome (ILAS) caused by *Klebsiella pneumoniae* (*K. pneumoniae*) is a newly emergent condition first described in 2012 by Enani and El-Khizzi [1], which incidence has significantly increased in the past ten years. We present the case of a 62-year-old Asian male who initially presented with a chief complaint of toothache but who was found to be in severe septic shock upon further assessment. Laboratory and imaging work revealed bilateral necrotizing *K. pneumoniae* pneumonia and multiple *Klebsiella* hepatic abscesses. A review of literature links the development of hepatic abscesses secondary to hepatobiliary or metastatic spread of either pulmonary or urinary tract infections [2-5]; however, to the best of our knowledge, this is the first reported case in which both pulmonary and hepatic abscess coexisted at the same time, and not as a consequence of the other, secondary to septic spread. We believe it is important to draw attention to this newly emergent syndrome, especially when leading to septic shock, as appropriate antibiotic duration and drainage of the abscesses are imperative for successful treatment and eradication of the infection.

## Case presentation

A 62-year-old Cantonese-speaking immigrant male with no reported past medical history, presented with a chief complaint of progressively worsening toothache for one month, and subjective fever and fatigue two days prior to presentation. A review of systems was significant for intermittent dry cough and blurry vision. In the emergency room, the patient was febrile (temperature 39.4 °C), hypotensive (blood pressure of 79/49 mmHg), tachypneic (respiratory rate of 28 breaths per minute), and hypoxic (oxygen saturation of 90% on room air). Laboratory work was significant for marked leukocytosis with left shift, increased procalcitonin and high sensitive C-reactive protein, normocytic anemia, acute kidney injury, hyponatremia, increased glucose level, and severely increased D-dimer level (Table 1).

**Table 1:** Initial laboratory values

Laboratory parameters	Patient's Values	Reference Range
WBC	34.33 x 10^3^/mcL	4.80 - 10.80 x 10^3^/mcL
Neutrophil %	89.9 %	44.0 - 70.0 %
Neutrophil Absolute	30.82 x 10^3^/mcL	2.10 - 7.60 x 10^3^/mcL
Immature Granulocytes Absolute	0.77 x 10^3^/mcL	0.00 - 0.20 x 10^3^/mcL
Hemoglobin	11.7 g/dL	14.0 - 18.0 g/dL
Hematocrit	34.5 %	42.0 - 52.0 %
MCV	81.4 fL	80.0 - 99.0 fL
RDW	13.1 %	12.0 - 15.0 %
Platelets	349 x 10^3^/mcL	150 - 450 x 10^3^/mcL
D-DIMER	31,391 ng/mL	0 - 243 ng/mL
Procalcitonin (PCT)	>100.00 ng/mL	0.02 - 0.10 ng/mL
BUN	28 mg/dL	8 - 22 mg/dL
Creatinine	1.67 mg/dL	0.50 - 1.30 mg/dL
eGFR	42 ml/min/1.73m^2^	>=60 ml/min/1.73m^2^
Glucose	519 mg/dL	65 - 115 mg/dL
Sodium	128 mmol/L	136 - 145 mmol/L
Potassium	4.7 mmol/L	3.5 - 5.0 mmol/L
Chloride	91 mmol/L	98 - 108 mmol/L
Bicarbonate	22 mmol/L	22 - 29 mmol/L
Calcium	7.9 mg/dL	8.6 - 10.2 mg/dL
Anion Gap	15 mEq/L	7 -16 mEq/L
Point of Care (POC) glucose capillary	458 mg/dL	65 - 115 mg/dL
High Sensitive C-Reactive Protein (CRP)	198.40 mg/L	0.00 - 5.00 mg/L

**Figure 1:**
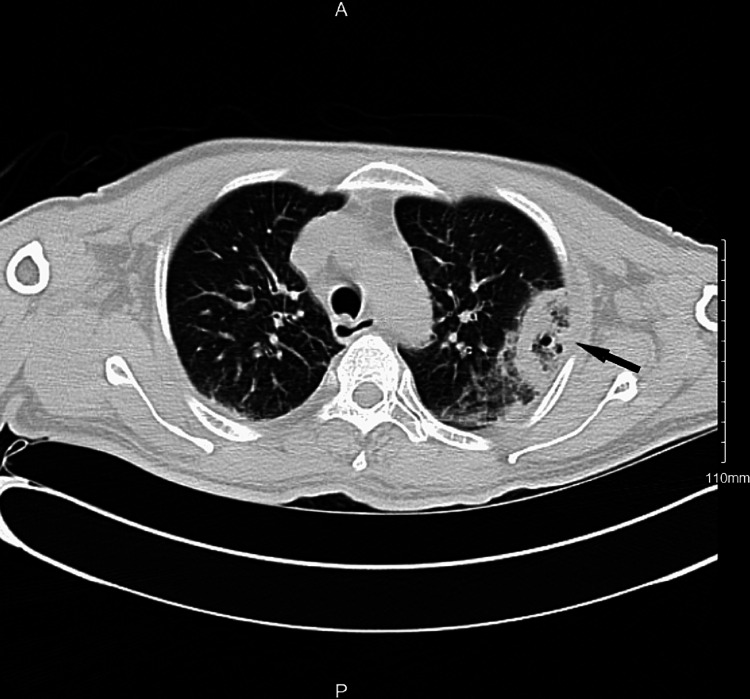
Computed tomography (CT) of chest depicting left upper lobe peripheral necrotizing lesion (arrow).

In view of tachypnea, hypoxia and elevated D-dimer level, the patient underwent computed tomography angiography (CTA) of the chest to rule out pulmonary embolism. No pulmonary embolism was detected; however, the patient was found to have bilateral pleural effusions associated with multiple necrotizing lesions on computed tomography (CT) of the chest (Figure 1).

The patient was initially admitted to the medical intensive care unit for management of septic shock secondary to bilateral necrotizing pneumonia and was started on vasopressor support and broad-spectrum antibiotic treatment with Meropenem.

**Figure 2:**
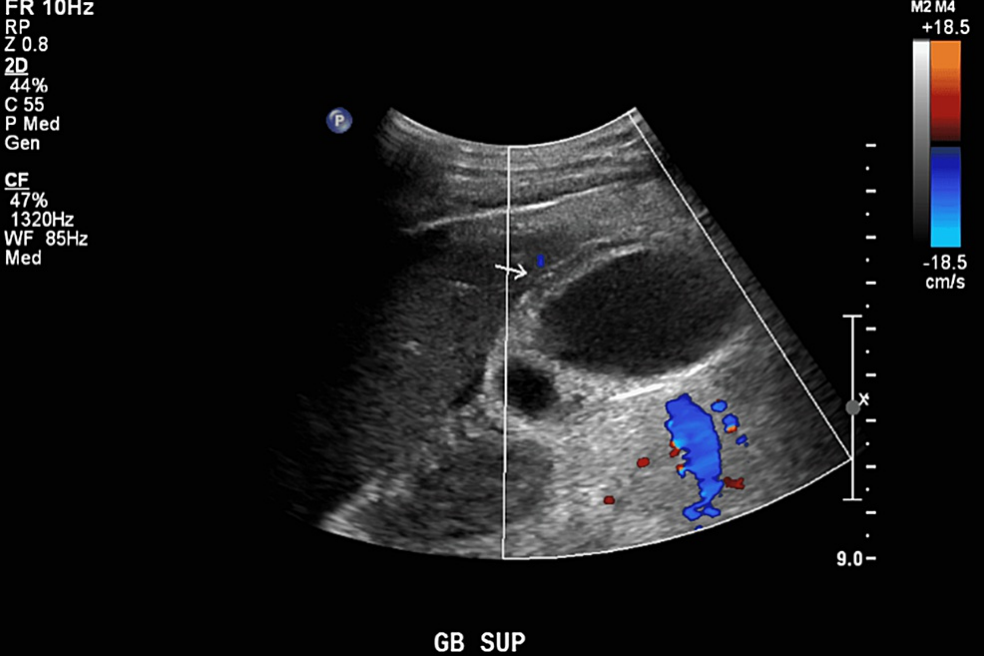
Ultrasound (US) of right upper quadrant depicting a distended gallbladder (arrow).

A partial view of the liver on CT of the chest also showed multiple mass-like hepatic lesions, prompting further evaluation of the abdomen. Abdominal ultrasound revealed multiple hepatic lesions concerning for metastasis versus abscesses as well as a distended gallbladder (Figure 2).

The CT of the abdomen and pelvis was suggestive of chronicity of the hepatic lesions, which were found to be cystic in nature and more suspicious for infectious rather than neoplastic etiology. Further laboratory work revealed the patient had prior hepatitis B infection, and severely uncontrolled diabetes mellitus (Table 2).

**Table 2:** Additional laboratory studies

Laboratory parameters	Patient's Values	Reference Range
Hemoglobin A1C	14.4 %	4.0 - 5.6 %
Hepatitis A IgG Antibody	Reactive	Nonreactive
Hepatitis B Surface Antigen	Nonreactive	Nonreactive
Hepatitis B Surface Antibody	Reactive	Nonreactive
Hepatitis B Core Antibody Total	Reactive	Nonreactive
Hepatitis B e Antigen	Negative	Negative
Hepatitis B e Antibody	Negative	Negative
Hepatitis C S/CO Ratio	0.10 S/CO	0.00 - 0.99 S/CO
Hepatitis C Interpretation	Nonreactive	Nonreactive

**Figure 3:**
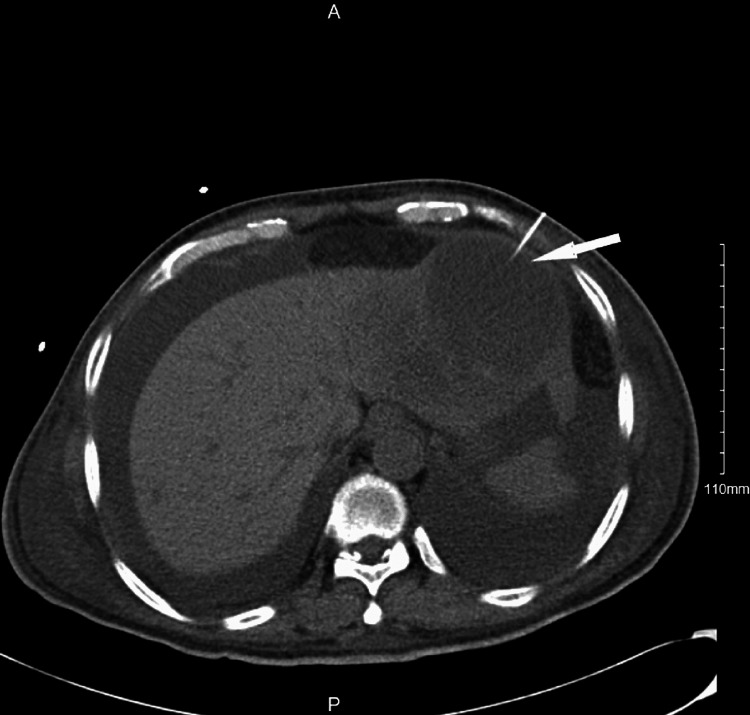
Computed tomography (CT) guided aspiration of hepatic cyst (arrow).

The Infectious Disease department was consulted, with recommendations to continue broad spectrum antibiotics with Meropenem and to drain the abscess in order to obtain abscess fluid studies. The patient subsequently underwent CT-guided drainage of the hepatic abscesses by Interventional Radiology (Figure 3).

The culture of the drainage fluid as well as blood cultures collected on admission were positive for *K. pneumoniae*. The Ophthalmology department was consulted as well in view of reported visual disturbances in the setting of newly diagnosed uncontrolled diabetes to evaluate for retinopathy, and to rule out endophthalmitis. An ophthalmologic exam was negative for endophthalmitis; however, it showed multiple cotton wool spots secondary to septic emboli on the bilateral eyes with foveal sparing.

The patient completed a total antibiotic course of six weeks and continues to follow up with the primary care outpatient clinic. Despite antibiotic therapy and negative repeat cultures, the patient continues to experience decreased visual acuity.

## Discussion

KPIS is a rare condition first described in 1980 in Taiwan [1], which incidence has significantly increased in the past ten years, and typically presents with hepatic involvement with abscesses formation, which is now recognized as an emerging syndrome called ILAS. Case reports of pyogenic liver abscesses have linked *K. pneumoniae* to the development of meningitis [2], endophthalmitis [3-5], otitis [6], spondylodiscitis [7], and pulmonary septic embolism [8]. To the best of our knowledge, we present the first case of multiorgan KPIS in the United States, in which at least three organ systems (pulmonary, gastrointestinal, and ophthalmic) were involved at the time of presentation. This report is also the first reported case in New York.

*K. pneumoniae* is a gram-negative bacterium, a member of the *Klebsiella* genus of *Enterobacteriaceae,* and belongs to the normal flora of the human mouth and intestine. Most community-acquired *K. pneumoniae* infections typically cause either pneumonia or urinary tract infections. However, rarely, it can also cause infections in other organ systems such as in the gastrointestinal tract, meninges, eyes, and ears. Most commonly, *K. pneumoniae* causes infection in alcoholic patients and those with diabetes, especially if uncontrolled, which was recently reported in at least three case reports linking diabetes and hepatic abscesses [1,8,9], and at least one case report linking diabetes and biliary abscesses [5].

There exist different polysaccharide capsular serotypes of *K. pneumoniae* which confer the bacterium different virulence properties. Expression of mucoviscosity-associated gene A (*magA*) and the regulator of mucoid phenotype A (*rmpA*) gene results in a hypermucoviscous phenotype, which has been linked with *K. pneumoniae* serotypes K1 and K2, the most common strains associated with KPIS [8,10]. Serotypes K1 and K2 are considered to be more virulent in nature, can infect either immunocompetent or immunocompromised hosts, and have been linked to the development of ILAS [11]. Studies have also shown that patients of southeast Asian and Asian heritage, especially those of Chinese descent, may be more prone to infection with this bacterium, as K1 and K2 serotypes are known colonizers of their gastrointestinal tract [12,13].

Particularly in New York, awareness of this emerging syndrome is important as New York City is a popular tourist destination and the home of many immigrant communities from all over the world, including one of the largest Asian and South East Asian populations in the country. However, it is important to recognize that this syndrome is not limited to patients of Asian or southeast Asian heritage but that it is also increasingly being reported in European countries [3] and other more geographically distanced countries such as Brazil [14], Saudi Arabia [1] and Australia [15]. Awareness of this new emergent syndrome will result in prompt recognition, proper treatment, prevention of further complications, and a decrease in the likelihood of development of resistance.

In the presented case, even though the patient completed the recommended six-week treatment with broad-spectrum antibiotics, and repeat blood cultures were negative, the patient experienced an increase in morbidity, characterized by permanent visual damage and prolonged hospital stay.

Several factors played a role in the acquisition of multiorgan KPIS by the presented patient. First, his uncontrolled diabetes mellitus made him prone to infections, especially by *K. pneumoniae*, increasing the likelihood of the development of complications such as hepatic pyogenic liver abscesses, necrotizing pneumonia, and bilateral ophthalmic septic emboli. Second, according to the case report by Lin et al. [13], the patient may have been at an increased risk of colonization by a hypervirulent *K. pneumoniae* strain given his country of origin. Third, although a link has not previously been documented, it is plausible that a prior hepatitis B infection could have caused hepatic damage which could have made the organ prone to subsequent insults. And fourth, health disparity likely played a role, as the patient presented in this case report was an immigrant who was uninsured and did not speak English, both of which factors likely contributed to the delay in presentation and extent of the disease process.

## Conclusions

This case report presented the case of a 62-year-old male of Chinese heritage who presented with septic shock secondary to multiorgan KPIS, characterized by the presence of bilateral necrotizing pneumonia, ILAS, and bilateral ophthalmic septic emboli. Despite receiving the recommended treatment, the patient experienced increased morbidity, requiring prolonged therapy, partially secondary to delay in presentation, which is presumed to have been the result of health disparities. It is important to bring light to this case as this emerging invasive syndrome caused by hypervirulent *K. pneumoniae *strains is being reported in an increasing number of countries, with this case report, to the best of our knowledge, being the first reported case in New York City, the home of one of the largest Asian population in the country and a frequent tourist destination. Early recognition of this syndrome could lead to fast and appropriate treatment and lead to the prevention of a further increase in morbidity and mortality.
